# Cutting Edge: Failure of Antigen-Specific CD4^+^ T Cell Recruitment to the Kidney during Systemic Candidiasis

**DOI:** 10.4049/jimmunol.1401675

**Published:** 2014-10-24

**Authors:** Rebecca A. Drummond, Carol Wallace, Delyth M. Reid, Sing Sing Way, Daniel H. Kaplan, Gordon D. Brown

**Affiliations:** * Aberdeen Fungal Group, Institute of Medical Sciences, University of Aberdeen, Aberdeen AB25 2ZD, United Kingdom;; † Division of Infectious Diseases, Cincinnati Children’s Hospital, Cincinnati, OH 45229; and; ‡ Department of Dermatology, Center for Immunology, University of Minnesota, Minneapolis, MN 55455

## Abstract

*Candida albicans* is the leading cause of systemic candidiasis, a fungal disease associated with high mortality and poor treatment options. The kidney is the target organ during infection and whose control is largely dependent on innate immunity, because lymphocytes appear redundant for protection. In this article, we show that this apparent redundancy stems from a failure of Ag-specific CD4^+^ T cells to migrate into infected kidneys. In contrast, Ag-specific CD8^+^ T cells are recruited normally. Using Ag-loaded immunoliposomes to artificially reverse this defective migration, we show that recruited Ag-specific CD4^+^ T cells polarize toward a Th17 phenotype in the kidney and are protective during fungal infection. Therefore, our data explain the redundancy of CD4^+^ T cells for defense against systemic infection with *C. albicans* and have important implications for our understanding of antifungal immunity and the control of renal infections.

## Introduction

*Candida albicans* is the leading cause of systemic candidiasis, a fungal bloodstream infection that has a high mortality, despite the availability of antifungal drugs (1). Systemic candidiasis occurs in a variety of manifestations, including infection of the kidney, which is commonly found in patients undergoing medical treatments, such as chemotherapy or gastrointestinal surgery. Systemic candidiasis can be modeled in mice by i.v. injection of yeast cells directly into the bloodstream. In this model, *C. albicans* primarily accumulates in the kidneys, which do not control fungal growth, whereas the infection is cleared in other organs, including the brain, spleen, and liver (2).

Immune control of renal *C. albicans* infections can be a predictor of host survival (3). The majority of studies analyzing *Candida* infections of the kidney concentrated on the role of neutrophils and macrophages, which are the major infiltrating leukocytes following infection and are necessary for host protection (2). Very little work has been done to understand the role of lymphocytes, particularly T cells, in renal *Candida* infections. T cells increase in this organ in a time-dependent manner following infection and are thought to play a protective role (2). However, there is substantial evidence that adaptive immunity is redundant for fungal control in the kidney. Notably, mice deficient in T cells, or in all lymphocytes, show no defect in controlling *C. albicans* infections in these tissues (4). In this study, we explored the reasons for this redundancy by characterizing *C. albicans*–specific T cells.

## Materials and Methods

### Mice

Eight- to twelve-week-old female C57BL/6 mice were bred at the Medical Research Facility at the University of Aberdeen. All experimentation conformed to U.K. Home Office regulations (60/4007) and the University of Aberdeen ethics committee.

### Adoptive transfers

Lymph nodes (LNs) and spleens were isolated from OT.I/OT.II donor mice and disaggregated through 70-μM filters, and cells were counted using trypan blue exclusion. CD4/8^+^ cells were negatively purified by depleting irrelevant populations using biotin-Ab mixture and anti-biotin MicroBeads (Miltenyi Biotec) and then stained with 5 μM CFSE (Invitrogen). A total of 3 × 10^6^ cells was injected i.v. into gender-matched recipient mice.

### Systemic candidiasis model

*C. albicans* strain Calb-Ag (5) was used for all experiments and was grown in YPD broth. Yeasts were washed and counted, and animals were infected i.v. with 2 × 10^5^ cells. Fungal burdens were determined by serial dilution on YPD agar.

### Abs and flow cytometry

The following Abs were used in this study: anti-CD3 (145-2C11), CD4 (GK1.5), Vα2 (B20.1), CD45.1 (A20), CD69 (H1.2F3), CD8 (53.6-7), CD25 (PC61), CD69 (H1.2F3), CD62L (MEL-14), CCR7 (4B12) (all from BD Biosciences); CD44 (IM7) (BioLegend); RORγt (B2D), T-bet (eBio4B10), Foxp3 (MF23), GATA-3 (TWAJ), CCR5 (7A4), CCR6 (sirx6), and CCR9 (CW-1.2) (all from eBioscience); CCR1 (643854), CCR2 (475301), CX3CR1 (polyclonal), and CXCR6 (221002) (all from R&D Systems); and CXCR4 (REA107) (Miltenyi Biotec). Abs were used at 0.5–1 μg/ml, and staining was performed with anti-CD16/32 (24G2). Intracellular staining was performed using the Foxp3 Staining Kit (eBioscience). Samples were acquired on a FACS LSR II cytometer (BD Biosciences). FlowJo software (TreeStar) was used for analysis.

### Isolation of renal leukocytes

Kidneys were digested with 0.08 mg/ml Liberase and 1 mg/ml DNase I (Roche) at 37°C and disaggregated through 70-μM filters, and RBCs were lysed (BD Pharm Lyse, BD Biosciences). Harvested cells were resuspended in 40% Percoll, overlaid onto 70% Percoll, and spun at 2000 rpm for 20 min at 20°C. Cells at the interphase were collected and analyzed by flow cytometry.

### MHC class II tetramer staining

Samples were incubated with 20 μg/ml PE-conjugated MHC class II tetramers bound with Als3 peptide (pAls3-Tet; S.S. Way, manuscript in preparation) for 60 min at room temperature. Samples were then labeled with anti-PE MicroBeads and purified using magnetic columns (Miltenyi Biotec). The resulting fractions were analyzed by flow cytometry.

### Generation of immunoliposomes

Liposomes were made, as previously described (6), and delivered as two i.v. doses with a 90-min gap between injections.

### Multiplex PCR

RNA was isolated from OT.II cells positively purified from the renal LNs using anti-CD45.1–biotin Ab and anti-biotin MicroBeads (Miltenyi Biotec), converted to cDNA using the SuperScript III synthesis system (Invitrogen), and used as a template in multiplex PCR reactions (Maxim Biotech), as per the manufacturers’ instructions.

### Chemokine array

Kidneys were homogenized in protease inhibitor (Roche), and 200 μg total protein was used to probe membranes conjugated with anti-chemokine detector Abs (R&D Systems).

### Statistics and data analysis

All data were analyzed with GraphPad Prism 5.04 using an unpaired two-tailed Student *t* test, with the exception of fungal burden data, for which a Mann–Whitney *U* test was used. Results were considered statistically significant at *p* < 0.05.

## Results and Discussion

### Ag-specific CD4^+^ T cells activate and divide in renal LNs

**FIGURE 1.**
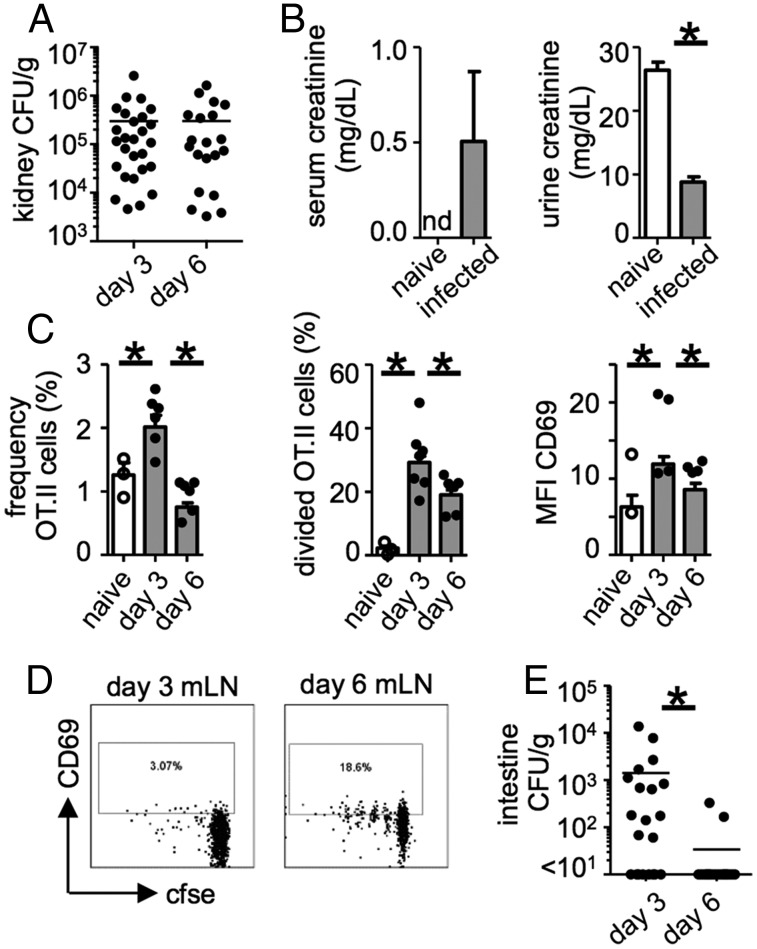
The Ag-specific CD4^+^ T cell response in the renal LNs becomes blunted during infection. A total of 3 × 10^6^ OT.II cells was adoptively transferred into WT mice, which were infected with 2 × 10^5^ CFU Calb-Ag 24 h later. Mice were analyzed for kidney fungal burdens (day 3, *n* = 39; day 6, *n* = 23) (**A**) and creatinine levels in the serum (naive *n* = 3, infected *n* = 5) and urine (naive *n* = 2, infected *n* = 8) (**B**). (**C**) Frequency (*left panel*; naive, *n* = 13; day 3, *n* = 27; day 6, *n* = 23), division as measured by CFSE dilution (*middle panel*; naive, *n* = 13; day 3, *n* = 27; day 6, *n* = 23), and CD69 expression (*right panel*; naive, *n* = 10; day 3, *n* = 19; day 6, *n* = 20) by OT.II cells in the renal LNs of naive and infected mice. (**D**) Example dot plots gated on OT.II cells (CD4^+^Vα2^+^CD45.1^+^) from mesenteric LNs. Plots are representative of ≥10 animals. (**E**) Intestine fungal burdens at day 3 (*n* = 21) and day 6 (*n* = 20) postinfection (data are from four pooled experiments). Bar charts show pooled data from four or five independent experiments; overlaid dot plots show data for one representative experiment. *p < 0.05. nd, not detected.

Because there are no TCR-transgenic models specific for *C. albicans*, we used a previously characterized strain, Calb-Ag, which expresses OVA and Eα peptides (5). This fungal strain enabled the use of transgenic OT.I/II and TEα cells to characterize Ag-specific T cell responses during systemic candidiasis. We adoptively transferred CD4^+^ OT.II cells into wild-type (WT) mice and infected these animals 24 h later with Calb-Ag. Systemic infection with Calb-Ag led to high fungal burdens in the kidney that were comparable at days 3 and 6 postinfection (Fig. 1A), similar to what we observed in animals infected with the parental strain (Supplemental Fig. 1A). Infection paralleled diminished renal function, because creatinine serum levels increased with reciprocally reduced urinary excretion among *C. albicans*–infected mice (Fig. 1B).

In the renal LNs, we found that the frequency of Ag-specific CD4^+^ OT.II cells did not correlate with kidney fungal burdens, which trended toward a negative relationship (Supplemental Fig. 1B). This was not seen in draining LNs of other infected tissues, such as the mesenteric LNs, in which the frequency of OT.II cells trended toward a positive relationship with intestinal fungal burdens (Supplemental Fig. 1C). Although there was an increase in the frequency, division, and activation of OT.II cells in the renal LN at day 3 postinfection, these parameters were all significantly reduced by day 6 (Fig. 1C), despite the similar fungal burdens at both time points (Fig. 1A). This reduction in T cell function was not evident in the mesenteric LNs; the division and activation of OT.II cells increased by day 6 (Fig. 1D) and correlated with a reduction in intestine fungal burdens (Fig. 1E). Thus, these data show that, in contrast to other tissues, Ag-specific CD4^+^ T cell responses in the renal LN become blunted during infection.

### Ag-specific CD4^+^ T cells do not migrate into infected kidneys

**FIGURE 2.**
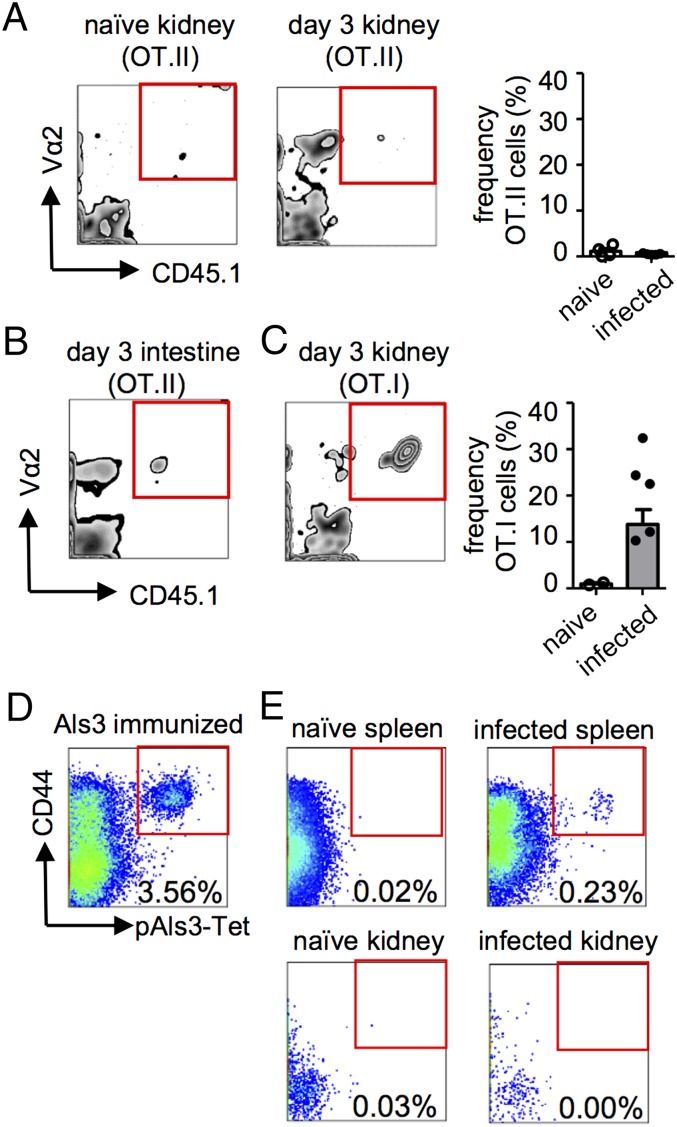
Ag-specific CD4^+^ T cells are not recruited to the infected kidney. Leukocytes from naive and infected kidneys (**A**) or small intestine (**B**) of mice, treated as in Fig. 1, were analyzed by flow cytometry for the presence of OT.II (Vα2^+^CD45.1^+^) cells. Example plots are gated on CD4^+^ lymphocytes and are representative of ≥12 animals from four experiments. Graph in (A) shows the frequency of OT.II cells in the kidney in naive (*n* = 4) and infected (*n* = 15) mice at day 3 postinfection. (**C**) Frequency of OT.I (Vα2^+^CD45.1^+^) cells in naive (*n* = 2) and infected (*n* = 10) kidneys at day 3 postinfection. Example plot is representative of 10 animals from two experiments and is gated on CD8^+^ lymphocytes. (**D**) pAls3-Tet staining of the draining LNs from an animal immunized with 20 μg recombinant Als3 in CFA 7 d postimmunization. (**E**) pAls3-Tet also was used to stain splenocytes (*upper panels*) and kidney-isolated leukocytes (*lower panels*) from infected mice at 7 d postinfection. Plots are representative of at least three animals from two independent experiments and are gated on B220^−^CD11b^−^CD11c^−^ CD3^+^CD4^+^CD8^−^ T cells. Data are pooled from two to four independent experiments.

We next characterized the recruitment of Ag-specific T cells into the kidney during infection. Unexpectedly, we could not detect Ag-specific CD4^+^ OT.II cells from *C. albicans*–infected kidneys at any time point analyzed (Fig. 2A, Supplemental Fig. 1D). In fact, the frequency of these cells was similar to naive controls (Fig. 2A). In contrast, OT.II cells were found in other infected tissues, such as the small intestine (Fig. 2B). Migration of TEα cells into the kidney was similarly defective (Supplemental Fig. 1E). In contrast to CD4^+^ T cells, Ag-specific CD8^+^ OT.I cells were robustly recruited to the infected kidney (Fig. 2C). Notably, unlike the OT.II response, the frequency of OT.I cells in the renal LNs showed a significant positive correlation with kidney fungal burdens (Supplemental Fig. 1F). This robust CD8 response shows that dendritic cell (DC) responses appear intact in this tissue during infection. Indeed, CX_3_CR1 DCs form a network throughout the renal interstitium (7) and, thus, are well positioned for capturing Ag as it enters the kidney.

To further verify our observations, we analyzed endogenous *C. albicans*–specific CD4^+^ T cells using pAls3-Tet. Als3 is a hyphae-expressed protein that is recognized by a major proportion of endogenous *Candida*-specific CD4^+^ T cells (8). These tetramers were functional, because high frequencies of pAls3-Tet^+^ T cells were detected in the draining LNs of animals immunized s.c. with recombinant Als3 protein in adjuvant (Fig. 2D). Moreover, pAls3-Tet^+^ T cells were detected in the spleen of *C. albicans*–infected animals (Fig. 2E). However, similar to our observations with the transgenic T cells, Als3-specific T cells were not detected in the infected kidney (Fig. 2E). Taken together, these data show that there is a defect in Ag-specific CD4^+^ T cell recruitment to the kidney during infection with *C. albicans*.

### Stimulated migration of Ag-specific CD4^+^ T cells protects the kidney against fungal infection

**FIGURE 3.**
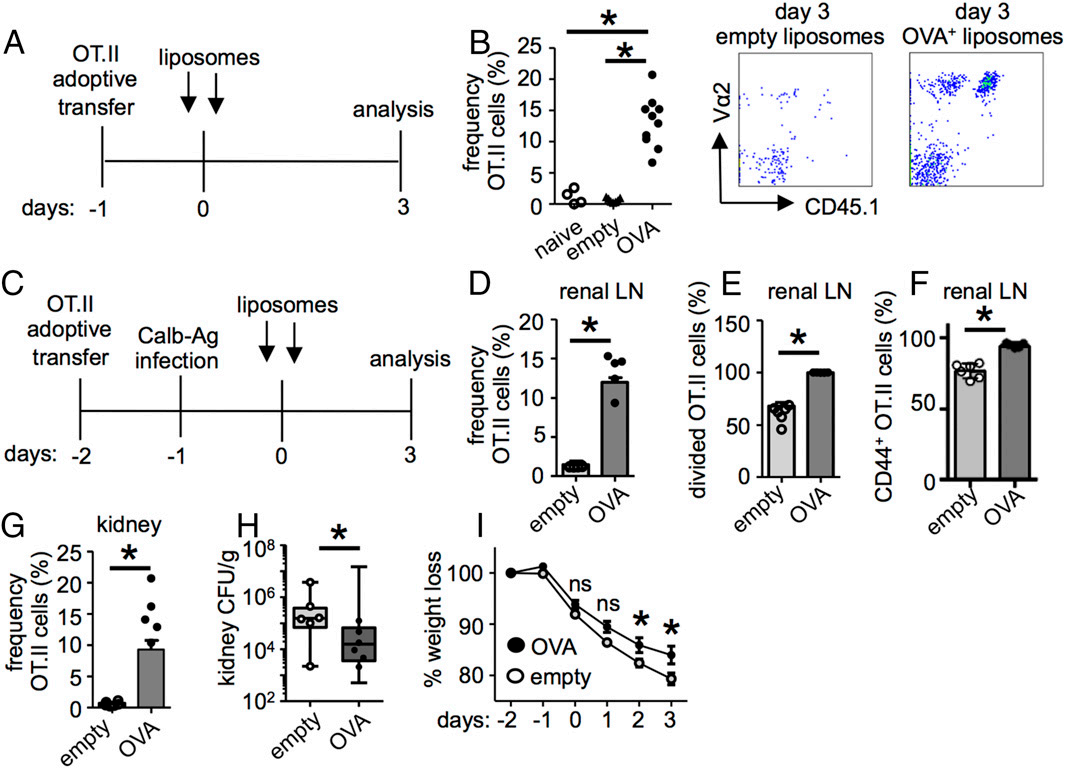
Ag-specific CD4^+^ T cells protect against infection. (**A**) A total of 3 × 10^6^ OT.II cells was adoptively transferred into WT mice, which were treated with either empty or OVA^+^ liposomes 24 h later. (**B**) Leukocytes isolated from the kidney at 3 d posttreatment were analyzed for the presence of OT.II cells (naive, *n* = 4; empty, *n* = 7; OVA, *n* = 10). Example plots are gated on CD4^+^ lymphocytes and are representative of at least six animals. (**C**) A total of 3 × 10^6^ OT.II cells was transferred to WT mice, which were then infected with 2 × 10^5^ CFU Calb-Ag and treated with either empty or OVA^+^ liposomes. The frequency (**D**), division (**E**), and activation (**F**) of OT.II cells in the renal LNs at day 3 postliposome treatment (empty, *n* = 12; OVA, *n* = 15). (**G**) The frequency of OT.II cells in the kidney (empty, *n* = 12; OVA, *n* = 15) at day 3 postliposome treatment. Kidney fungal burdens (**H**) and weight loss (**I**) in infected mice treated with either empty liposomes (*n* = 12) or OVA^+^ liposomes (*n* = 19). Bar charts show pooled data from two or three independent experiments; overlaid dot plots show data for one representative experiment. *p < 0.05. ns, not significant.

To artificially stimulate Ag-specific CD4^+^ T cell migration into the kidney during fungal infection, we made use of OVA-loaded immunoliposomes (OVA^+^ liposomes). These liposomes are conjugated to an Ab against α8-integrin, which targets Ag to the glomeruli (6). To test this system, mice were treated with OVA^+^ liposomes 24 h after receiving OT.II cells, and the kidneys were analyzed 3 d later for OT.II recruitment (Fig. 3A). Treatment with OVA^+^ liposomes led to high frequencies of OT.II T cells in these organs (Fig. 3B), and it also enhanced numbers in the gut (Supplemental Fig. 1G). In contrast, there was no OT.II recruitment to the kidneys of mice treated with liposomes lacking OVA (Fig. 3B).

We next examined the effect of these liposomes on CD4^+^ T cell responses in the kidney during infection. For these experiments, we transferred OT.II cells into recipient mice, infected them with Calb-Ag, and then treated the animals with OVA^+^ or empty liposomes (Fig. 3C). Treatment of infected mice with OVA^+^ liposomes significantly increased the frequency (Fig. 3D), division (Fig. 3E), and activation (Fig. 3F, Supplemental Fig. 2A) of OT.II cells in the renal LNs compared with infected animals given empty liposomes. OVA^+^ liposome treatment also induced recruitment of OT.II cells into the kidneys of infected mice (Fig. 3G). The ability of liposomes to drive OT.II cells into the kidney suggests that the defect in recruitment during normal infection is not due to an inhibitory factor produced by *C. albicans*. Notably, treatment with OVA^+^ liposomes led to significantly reduced kidney fungal burdens (Fig. 3H) and reduced weight loss of infected animals (Fig. 3I), but it did not affect intestinal fungal burdens (Supplemental Fig. 1H). Thus, these data show that Ag-specific CD4^+^ T cells can facilitate clearance of renal *C. albicans* infections.

### Protective Ag-specific CD4^+^ T cells have a Th17 phenotype and specific chemokine receptor expression profile

**FIGURE 4.**
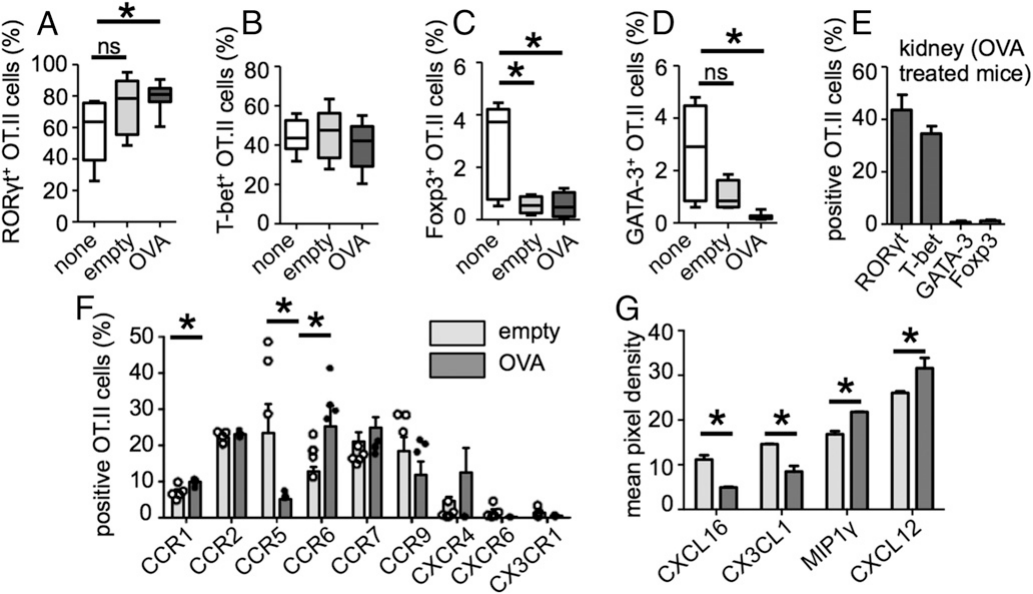
OVA^+^ liposomes enhance Th17 polarization in infected mice. Mice treated as in Fig. 3C (or left untreated; none) were analyzed for expression of master transcription factors RORγt (**A**), T-bet (**B**), Foxp3 (**C**), and GATA-3 (**D**) in the renal LNs by intracellular staining followed by flow cytometry. Data for RORγt/T-bet staining are pooled from three experiments (untreated, *n* = 9; empty, *n* = 12; OVA, *n* = 18), and data for Foxp3/GATA-3 staining are pooled from two experiments (untreated, *n* = 7; empty, *n* = 6; OVA, *n* = 10). (**E**) Expression of indicated transcription factors by OT.II cells in the kidney of OVA^+^ liposome–treated mice (*n* = 11). (**F**) Expression of indicated chemokine receptors by OT.II cells in the renal LNs, as determined by flow cytometry. (**G**) Relative expression of indicated chemokines, as determined by Western blot. *p < 0.05. ns, not significant.

We next explored how OVA^+^ liposomes affected the polarization of Ag-specific T cells, because the manipulation of T cell polarization can have profound consequences for control of *Candida* infection (1). For these experiments, we analyzed the expression of master transcription factors by OT.II cells in the renal LNs and kidney of infected mice, following treatment with either OVA^+^ liposomes or empty liposomes. We found that the predominant transcription factors expressed by OT.II cells in infected mice were T-bet and RORγt, which are indicative of Th1 and Th17 polarization, respectively (Fig. 4A, 4B, Supplemental Fig. 2B). OVA^+^ liposomes significantly enhanced RORγt expression by OT.II cells in the renal LNs compared with untreated mice (Fig. 4A), but they did not affect expression of T-bet (Fig. 4B). Furthermore, OVA^+^ liposomes caused significantly reduced expression of Foxp3 and GATA-3 by OT.II cells (indicative of regulatory T cells and Th2 cells, respectively). Empty liposomes had similar effects, but these were less consistent (Fig. 4). OT.II cells in the kidney could only be analyzed in the OVA^+^ liposome–treated mice, which had sufficient recruited cells for accurate analysis. Similar to the renal LNs, recruited OT.II cells were predominantly RORγt^+^ and T-bet^+^ (Fig. 4E). Th17 cells and IL-17 signaling are crucial for defense against systemic infection (1). Our data show that OVA^+^ liposomes specifically enhance Th17 polarization of Ag-specific CD4^+^ T cells.

We next characterized the expression of chemokine receptors in the liposome-treated mice. Multiplex PCR on cDNA generated from Ag-specific CD4^+^ T cells purified from the renal LNs of infected mice treated either with empty or OVA^+^ liposomes revealed differential regulation of several receptors in these lymphocytes (Supplemental Fig. 2C). These differences could be recapitulated by flow cytometry, which revealed enhanced expression of CCR1 and CCR6, but reduced CCR5, by OT.II cells in OVA^+^ liposome–treated animals (Fig. 4F). CCR6 was shown to mediate the migration of Th17 cells into nephritic kidneys (9), whereas CCR5 is associated with immunopathology in this tissue (10).

We also analyzed chemokine expression in the kidney by Western blot array (Supplemental Fig. 2D). Although CXCL16 and CX3CL1 (fractalkine) were increased significantly in empty liposome–treated kidneys (Fig. 4G), MIP1γ and CXCL12 were significantly reduced (Fig. 4G). This suggests that, despite the enhanced levels of some chemokines during infection, important signals remain missing. Therefore, our data show that OVA^+^ liposome treatment stimulates the expression of a distinct profile of chemokine receptors and ligands, inducing Ag-specific T cell migration into the kidney.

In conclusion, we discovered that there is defective recruitment of Ag-specific CD4^+^ T cells to the kidney during systemic *C. albicans* infection. Although further work is needed to understand the reasons for this deficiency, as well as why it does not affect CD8^+^ T cells, our observations revealed why the adaptive immune system is redundant during these infections. Moreover, we showed that artificially restoring CD4^+^ T cell responses in the kidney improves infection outcome. These findings have significant implications for our understanding of antifungal immunity and control of fungal growth within the kidney.

## Supplementary Material

Data Supplement
