# Supplementary material for: Cutting Edge: Failure of Antigen-Specific CD4+ T Cell Recruitment to the Kidney during Systemic Candidiasis
Source: J Immunol. 2014 Oct 24;193(11):5381–5. doi: 10.4049/jimmunol.1401675 (PMC4238746; doi:10.4049/jimmunol.1401675)
Supplement: Data Supplement [file supp_193_11_5381__index.html]

Cutting Edge: Failure of Antigen-Specific CD4+ T Cell Recruitment to the Kidney during Systemic Candidiasis — Data Supplement 

# Cutting Edge: Failure of Antigen-Specific CD4+ T Cell Recruitment to the Kidney during Systemic Candidiasis

## Data Supplement

**Files in this Data Supplement:**

- Supplemental Figures 1 (PDF)
