# Supplementary material for: Cutting Edge: Failure of Antigen-Specific CD4+ T Cell Recruitment to the Kidney during Systemic Candidiasis
Source: J Immunol. 2014 Oct 24;193(11):5381–5. doi: 10.4049/jimmunol.1401675 (PMC4238746; doi:10.4049/jimmunol.1401675)
Supplement: Data Supplement [file 1401675_JI_1401675_Supplemental_Figures_1.pdf]

# FIGURE S1

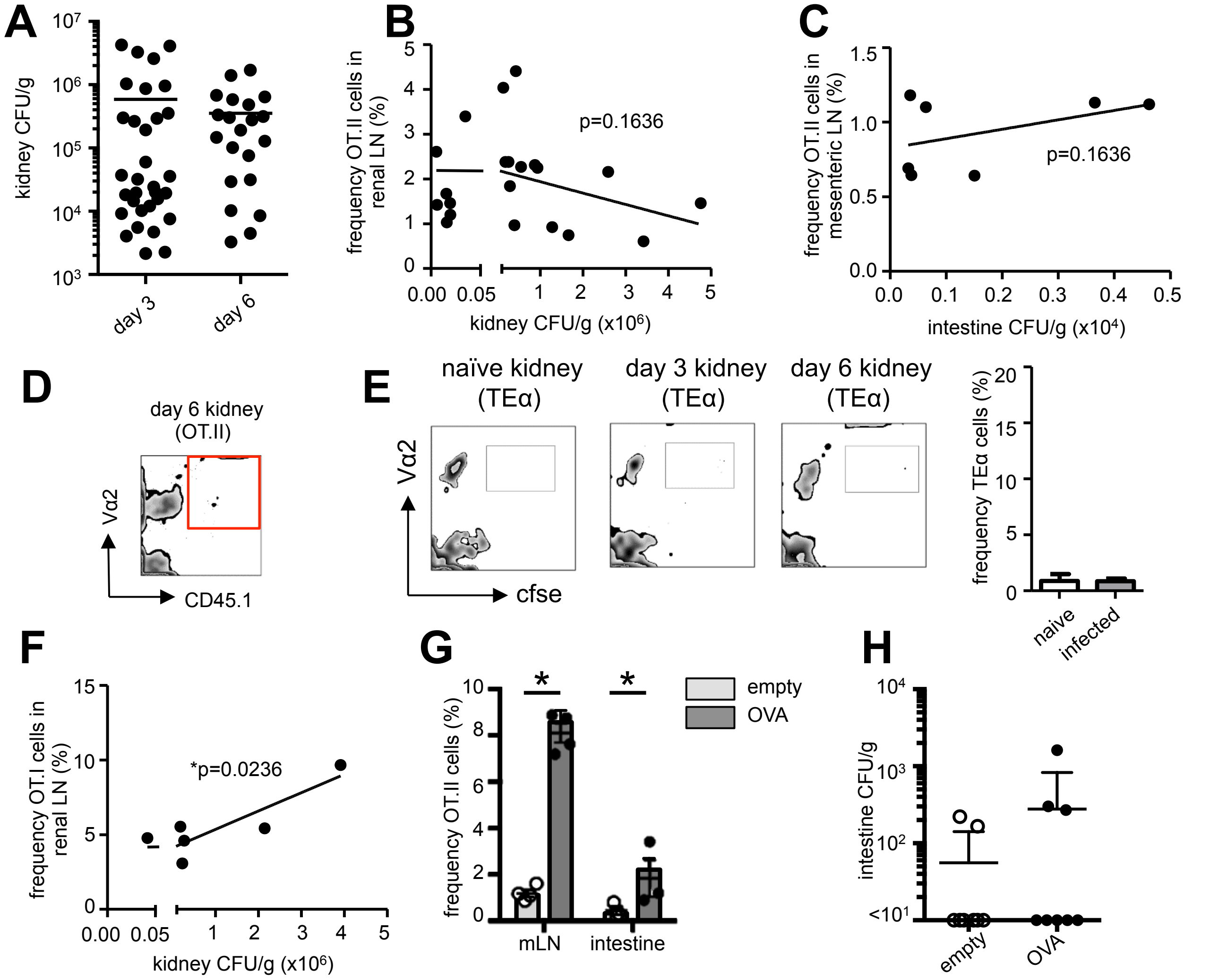


**Supplemental Figure 1**

(A) Kidney fungal burdens in animals infected with SC5314 and did not receive an OT.II adoptive transfer prior to infection (n=21-32); pooled from 4 experiments. (B) Frequency of $CD4^+$ OT.II cells in the renal LN at day 3 post-infection was measured by flow cytometry, and plotted against kidney fungal burdens (n=21). (C) Frequency of $CD4^+$ OT.II cells in the mesenteric LN at day 3 post-infection was measured by flow cytometry, and plotted against intestine fungal burdens (n=7). (D) Leukocytes from day 6 infected kidneys of mice treated as in Figure 1 were analysed by flow cytometry for the presence of OT.II ($V\alpha2^+CD45.1^+$) cells. Example plot is gated on $CD4^+$ lymphocytes and is representative of at least 12 animals from 4 experiments. (E) WT mice were adoptively transferred with $3x10^6$ $CD4^+$ TEα cells, infected with $2x10^5$ CFU Calb-Ag and analysed at day 3 post-infection. Leukocytes in the kidney were analysed by flow cytometry for the presence of TEα cells ($V\alpha2^+V\beta6^+CFSE^+$). Example plots are representative of at least 2 animals from two experiments, and are gated on $CD4^+V\beta6^+$ lymphocytes. Graph shows the frequency of TEα cells in the kidney in naïve (n=2) and infected (n=7) mice. (F) Frequency of $CD8^+$ OT.I cells in the renal LN at day 3 post-infection was measured by flow cytometry, and plotted against kidney fungal burdens (n=6). (G) Frequency of OT.II cells in indicated tissues and (H) intestine fungal burdens in animals at 3 days post-infection that had been treated with either empty or $OVA^+$ liposomes. Data in (G) from two pooled experiments, data in (H) from one experiment. *p<0.05 by unpaired t-test.

# FIGURE S2

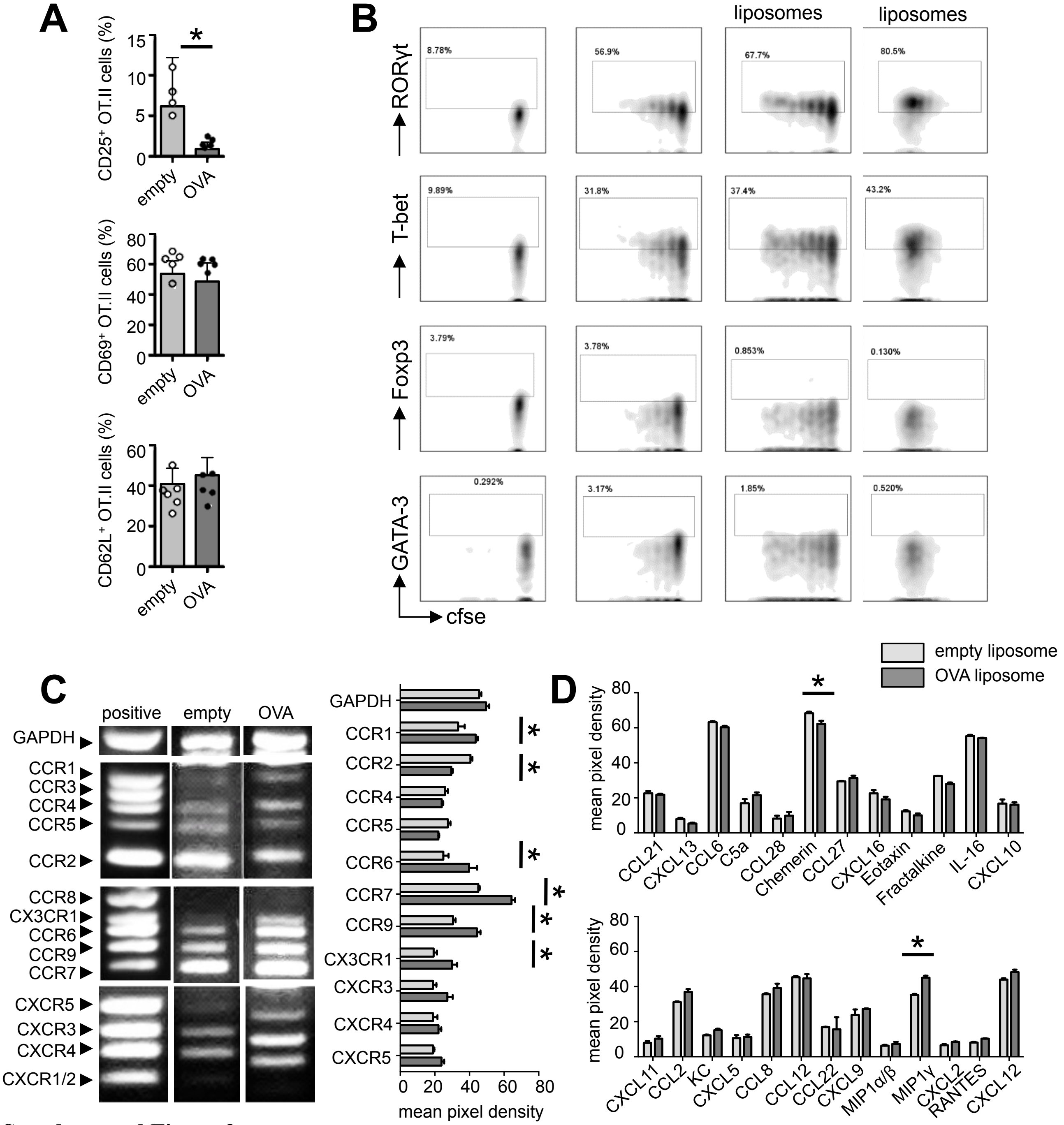


**Supplemental Figure 2**

(A) Expression of indicated activation markers by OT.II cells in the renal LNs of infected mice treated with empty or OVA liposomes, as determined by flow cytometry. Bar charts show pooled data (2 experiments, n=10), dot plots show a single representative experiment. (B) Example intracellular staining for indicated master transcription factors expressed by OT.II cells in the renal LN of infected mice at 3 days post-liposome treatment. Plots are representative of at least 6 animals from 3 independent experiments, and are gated on OT.II cells ($CD4^+V\alpha2^+CD45.1^+$). (C) Multiplex PCR for indicated genes on $CD45.1^+$ cells purified from the renal LN of infected mice treated with empty or OVA liposomes. PCR was performed on equal amounts of cDNA and analysed after 30 amplification cycles. Mean pixel densities are given as an approximate quantification guide. (D) Quantification of indicated chemokines in whole kidney homogenates as determined by Western Blot. Data in (C) and (D) are from a single experiment.
